# Supplementary material for: Canine Mesenchymal-Stem-Cell-Derived Extracellular Vesicles Attenuate Atopic Dermatitis
Source: Animals (Basel). 2023 Jul 6;13(13):2215. doi: 10.3390/ani13132215 (PMC10339941; doi:10.3390/ani13132215)
Supplement: Supplementary file 1 [file animals-13-02215-s001.zip › animals-2425301-supplementary.pdf]

animals

MDPI

Supplementary Materials

Table S1-1. Hematological values of male mice in the 28-day repeat-dose toxicity study.

Test items	Group G1 (0)	G2 (7.45E+08)	G3 (2.98E+09)	G4 (1.19E+10)
**WBC[1] (K/μL)**	**2.29 ± 1.18 (10)**	**3.78 ± 3.39 (10)**	**2.29 ± 1.16** (10)	2.02 ± 1.16 (10)
NE[2] (K/μL)	0.27 ± 0.10 (10)	0.36 ± 0.26 (10)	0.23 ± 0.06 (10)	0.24 ± 0.12 (10)
EO[3] (K/μL)	0.07 ± 0.09 (10)	0.26** ± 0.15 (10)	0.21** ± 0.14 (10)	0.10 ± 0.07 (10)
BA[4] (K/μL)	0.00 ± 0.00 (10)	0.00 ± 0.00 (10)	0.00 ± 0.00 (10)	0.00 ± 0.00 (10)
LY[5] (K/μL)	1.89 ± 1.05 (10)	3.07 ± 2.96 (10)	1.80 ± 1.03 (10)	1.64 ± 1.01 (10)
MO[6] (K/μL)	0.04 ± 0.02 (10)	0.06 ± 0.04 (10)	0.04 ± 0.03 (10)	0.03 ± 0.02 (10)
LUC[7] (K/μL)	0.01 ± 0.01 (10)	0.02 ± 0.02 (10)	0.01 ± 0.01 (10)	0.01 ± 0.01 (10)
NEP[8] (%)	13.2 ± 3.9 (10)	11.1 ± 3.2 (10)	11.4 ± 4.1 (10)	12.4 ± 3.2 (10)
EOP[9] (%)	2.7 ± 1.4 (10)	8.2** ± 3.7 (10)	9.2** ± 4.9 (10)	5.9 ± 3.5 (10)
BAP[10] (%)	0.1 ± 0.1 (10)	0.0 ± 0.0 (10)	0.0 ± 0.1 (10)	0.2 ± 0.2 (10)
LYP[11] (%)	81.9 ± 3.9 (10)	78.7 ± 5.8 (10)	77.5 ± 5.8 (10)	79.7 ± 3.8 (10)
MOP[12] (%)	1.8 ± 1.0 (10)	1.5 ± 0.7 (10)	1.5 ± 0.7 (10)	1.2 ± 0.5 (10)
LUP[13] (%)	0.4 ± 0.1 (10)	0.5 ± 0.2 (10)	0.4 ± 0.1 (10)	0.6* ± 0.2 (10)
RBC[14] (M/μL)	8.12 ± 0.38 (10)	8.19 ± 0.40 (10)	7.59** ± 0.30 (10)	7.91 ± 0.44 (10)
Hb[15] (g/dℓ)	13.2 ± 0.5 (10)	13.1 ± 0.9 (10)	12.4* ± 0.6 (10)	12.9 ± 0.5 (10)
RDW[16] (%)	11.9 ± 0.5 (10)	11.9 ± 0.4 (10)	12.2 ± 0.6 (10)	12.2 ± 0.5 (10)
HCT[17] (%)	44.2 ± 1.6 (10)	45.0 ± 1.9 (10)	41.9** ± 1.4 (10)	43.5 ± 2.2 (10)
MCV[18] (fL)	54.6 ± 2.9 (10)	55.1 ± 2.4 (10)	55.2 ± 1.7 (10)	55.1 ± 2.0 (10)
MCH[19] (pg)	16.3 ± 0.6 (10)	16.0 ± 0.9 (10)	16.3 ± 0.7 (10)	16.4 ± 0.4 (10)
MCHC[20] (g/dℓ)	29.8 ± 0.7 (10)	29.1 ± 1.5 (10)	29.6 ± 1.1 (10)	29.7 ± 0.8 (10)
Reti[21] (%)	3.80 ± 0.50 (10)	3.34 ± 0.36 (10)	3.43 ± 0.40 (10)	3.65 ± 0.47 (10)
PLT[22] (K/μL)	1349 ± 98 (10)	1291 ± 149 (10)	1171 ± 140 (10)	1244 ± 199 (10)
MPV[23] (fL)	7.4 ± 0.7 (10)	7.5 ± 0.2 (10)	7.3 ± 0.5 (10)	7.5 ± 0.5 (10)

Mean ± S.D (number of animals). 1: white blood cell count, 2: neutrophil, 3: eosinophil, 4: basophil, 5: lymphocyte, 6: monocyte, 7: large unstained cell, 8: percent of neutrophil, 9: percent of eosinophil, 10: percent of basophil, 11: percent of lymphocyte, 12: percent of monocyte, 13: percent of the large unstained cell, 14: red blood cell count, 15: hemoglobin conc., 16: red cell distribution width, 17: hematocrit, 18: mean corpuscular volume, 19: mean corpuscular hemoglobin, 20: mean corpuscular hemoglobin concentration, 21: reticulocyte, 22: platelet, 23: mean platelet volume. *: Significant difference compared to the control group value; $p < 0.05$. **: Significant difference compared to the control group value; $p < 0.01$.

Table S1-2. Hematological values of female mice in the 28-day repeat-dose toxicity study.

Test items	Group G1 (0)	G2 (7.45E+08)	G3 (2.98E+09)	G4 (1.19E+10)
**WBC[1] (K/μL)**	**2.70 ± 1.12 (10)**	**3.11 ± 1.34 (10)**	**3.25 ± 0.96** (10)	2.83 ± 1.32 (10)
NE[2] (K/μL)	0.34 ± 0.16 (10)	0.31 ± 0.13 (10)	0.42 ± 0.11 (10)	0.34 ± 0.15 (10)
EO[3] (K/μL)	0.12 ± 0.10 (10)	0.29* ± 0.13 (10)	0.23* ± 0.09 (10)	0.23* ± 0.14 (10)
BA[4] (K/μL)	0.00 ± 0.00 (10)	0.00 ± 0.00 (10)	0.00 ± 0.00 (10)	0.00 ± 0.00 (10)
LY[5] (K/μL)	2.17 ± 1.01 (10)	2.42 ± 1.14 (10)	2.51 ± 0.80 (10)	2.18 ± 1.05 (10)
MO[6] (K/μL)	0.06 ± 0.03 (10)	0.07 ± 0.04 (10)	0.07 ± 0.03 (10)	0.06 ± 0.03 (10)
LUC[7] (K/μL)	0.02 ± 0.01 (10)	0.02 ± 0.01 (10)	0.04* ± 0.02 (10)	0.02 ± 0.01 (10)
NEP[8] (%)	12.7 ± 4.4 (10)	10.2 ± 1.6 (10)	13.2 ± 2.9 (10)	12.4 ± 3.1 (10)
EOP[9] (%)	5.5 ± 6.8 (10)	10.0 ± 4.0 (10)	7.0 ± 1.9 (10)	8.0 ± 2.5 (10)
BAP[10] (%)	0.1 ± 0.1 (10)	0.1 ± 0.1 (10)	0.1 ± 0.1 (10)	0.1 ± 0.1 (10)
LYP[11] (%)	79.0 ± 5.4 (10)	77.0 ± 4.9 (10)	76.5 ± 4.1 (10)	76.9 ± 4.7 (10)
MOP[12] (%)	2.2 ± 1.3 (10)	2.0 ± 1.3 (10)	2.1 ± 0.8 (10)	2.0 ± 0.5 (10)
LUP[13] (%)	0.6 ± 0.3 (10)	0.6 ± 0.3 (10)	1.1 ± 0.7 (10)	0.7 ± 0.2 (10)
RBC[14] (M/μL)	8.34 ± 0.27 (10)	8.07 ± 0.52 (10)	8.07 ± 0.36 (10)	8.29 ± 0.35 (10)

Hb[15] (g/dℓ)	13.8 ± 0.4 (10)	13.6 ± 0.8 (10)	13.3 ± 0.4 (10)	13.7 ± 0.5 (10)
RDW[16] (%)	12.2 ± 0.7 (10)	12.4 ± 0.5 (10)	12.6 ± 0.4 (10)	12.6 ± 0.6 (10)
HCT[17] (%)	45.1 ± 1.6 (10)	44.4 ± 2.1 (10)	44.6 ± 1.7 (10)	45.9 ± 2.3 (10)
MCV[18] (fL)	54.1 ± 1.7 (10)	55.1 ± 1.7 (10)	55.4 ± 1.9 (10)	55.3 ± 1.0 (10)
MCH[19] (pg)	16.6 ± 0.4 (10)	16.9 ± 0.4 (10)	16.6 ± 0.4 (10)	16.6 ± 0.3 (10)
MCHC[20] (g/dℓ)	30.7 ± 1.0 (10)	30.6 ± 0.8 (10)	30.0 ± 0.6 (10)	29.9 ± 0.7 (10)
Reti[21] (%)	3.08 ± 1.42 (10)	3.01 ± 1.23 (10)	3.86 ± 0.89 (10)	4.05 ± 0.98 (10)
PLT[22] (K/μL)	1103 ± 213 (10)	1133 ± 322 (10)	1120 ± 126 (10)	1095 ± 159 (10)
MPV[23] (fL)	7.0 ± 0.4 (10)	7.2 ± 0.5 (10)	7.5* ± 0.6 (10)	7.7* ± 0.4 (10)

Mean ± S.D (number of animals). 1: white blood cell count, 2: neutrophil, 3: eosinophil, 4: basophil, 5: lymphocyte, 6: monocyte, 7: large unstained cell, 8: percent of neutrophil, 9: percent of eosinophil, 10: percent of basophil, 11: percent of lymphocyte, 12: percent of monocyte, 13: percent of the large unstained cell, 14: red blood cell count, 15: hemoglobin conc., 16: red cell distribution width, 17: hematocrit, 18: mean corpuscular volume, 19: mean corpuscular hemoglobin, 20: mean corpuscular hemoglobin concentration, 21: reticulocyte, 22: platelet, 23: mean platelet volume. *: Significant difference compared to the control group value; $p < 0.05$.

Table S2-1. Blood chemistry values for male mice in the 28-day repeat-dose toxicity study.

**Test items**	**Group**			
	**G1 (0)**	**G2 (7.45E+08)**	**G3 (2.98E+09)**	**G4 (1.19E+10)**
**AST[1] (IU/L)**	**53 ± 9 (10)**	**57 ± 6 (10)**	**61 ± 23** (10)	51 ± 7 (10)
ALT[2] (IU/L)	41 ± 10 (10)	42 ± 8 (10)	56 ± 33 (10)	37 ± 6 (10)
ALP[3] (IU/L)	222 ± 35 (10)	227 ± 45 (10)	231 ± 33 (10)	230 ± 74 (10)
BIL[4] (mg/dℓ)	0.15 ± 0.04 (10)	0.16 ± 0.03 (10)	0.18 ± 0.0 (10)	0.18 ± 0.1 (10)
BUN[5] (mg/dℓ)	26.8 ± 4.0 (10)	28.6 ± 4.4 (10)	24.9 ± 3.9 (10)	25.0 ± 2.9 (10)
CRE[6] (mg/dℓ)	0.3 ± 0.0 (10)	0.3 ± 0.0 (10)	0.3 ± 0.0 (10)	0.3 ± 0.1 (10)
UA[7] (mg/dℓ)	1.4 ± 0.9 (10)	1.2 ± 0.6 (10)	1.7 ± 1.1 (10)	1.5 ± 0.8 (10)
GLU[8] (mg/dℓ)	201 ± 45 (10)	217 ± 77 (10)	203 ± 54 (10)	197 ± 54 (10)
CHO[9] (mg/dℓ)	146 ± 22 (10)	155 ± 19 (10)	136 ± 15 (10)	170 ± 41 (10)
TG[10] (mg/dℓ)	89 ± 35 (10)	129 ± 53 (10)	108 ± 75 (10)	113 ± 90 (10)
PRO[11] (g/dℓ)	4.9 ± 0.2 (10)	4.9 ± 0.1 (10)	4.8* ± 0.1 (10)	5.1 ± 0.2 (10)
ALB[12] (g/dℓ)	1.7 ± 0.1 (10)	1.7 ± 0.1 (10)	1.7 ± 0.1 (10)	1.7 ± 0.1 (10)
A/G ratio[13]	0.52 ± 0.01 (10)	0.53 ± 0.02 (10)	0.52 ± 0.02 (10)	0.52 ± 0.02 (10)
LDH[14] (IU/L)	662 ± 354 (10)	621 ± 312 (10)	554 ± 232 (10)	572 ± 289 (10)
CPK[15] (U/L)	132 ± 87 (10)	164 ± 63 (10)	109 ± 30 (10)	107 ± 39 (10)
Ca[16] (mg/dℓ)	9.1 ± 0.3 (10)	9.0 ± 0.4 (10)	9.0 ± 0.4 (10)	9.2 ± 0.6 (10)
IP[17] (mg/dℓ)	8.2 ± 1.9 (10)	8.5 ± 1.8 (10)	7.7 ± 1.5 (10)	8.7 ± 2.1 (10)
Mg[18] (mg/dℓ)	3.1 ± 0.3 (10)	3.0 ± 0.4 (10)	2.9 ± 0.3 (10)	3.1 ± 0.4 (10)
Na[19] (mmol/L)	161 ± 4 (10)	161 ± 3 (10)	159 ± 2 (10)	160 ± 4 (10)
K[20] (mmol/L)	5.4 ± 0.7 (10)	5.3 ± 0.5 (10)	5.0 ± 0.5 (10)	5.2 ± 0.7 (10)
Cl[21] (mmol/L)	113 ± 1 (10)	113 ± 2 (10)	115** ± 3 (10)	116** ± 1 (10)

Mean ± S.D (number of animals). 1: aspartate aminotransferase, 2: alanine aminotransferase, 3: alkaline phosphatase, 4: total bilirubin, 5: blood urea nitrogen, 6: creatinine, 7: uric acid, 8: glucose, 9: total cholesterol, 10: triglyceride, 11: total protein, 12: albumin, 13: albumin/globulin ratio, 14: lactate dehydrogenase, 15: creatine phosphokinase, 16: calcium, 17: inorganic phosphorus, 18: magnesium, 19: sodium, 20: potassium, 21: chloride. *: Significant difference compared to the control group value; $p < 0.05$. **: Significant difference compared to the control group value; $p < 0.01$.

Table S2-2. Blood chemistry values for female mice in the 28-day repeat-dose toxicity study.

**Test items**	**Group**			
	**G1 (0)**	**G2 (7.45E+08)**	**G3 (2.98E+09)**	**G4 (1.19E+10)**
AST[1] (IU/L)	54 ± 9 (10)	51 ± 6 (10)	52 ± 5 (10)	60 ± 16 (10)
ALT[2] (IU/L)	33 ± 9 (10)	29 ± 5 (10)	33 ± 7 (10)	38 ± 19 (10)
ALP[3] (IU/L)	269 ± 67 (10)	245 ± 52 (10)	246 ± 32 (10)	238 ± 55 (10)
BIL[4] (mg/dℓ)	0.06 ± 0.03 (10)	0.05 ± 0.02 (10)	0.06 ± 0.0 (10)	0.06 ± 0.0 (10)
BUN[5] (mg/dℓ)	19.9 ± 3.3 (10)	19.3 ± 3.2 (10)	17.7 ± 3.5 (10)	20.5 ± 4.7 (10)

CRE[6] (㎎/dℓ)	0.3 ± 0.1 (10)	0.3 ± 0.0 (10)	0.3 ± 0.0 (10)	0.3 ± 0.1 (10)
UA[7] (㎎/dℓ)	1.0 ± 0.6 (10)	0.9 ± 0.4 (10)	0.9 ± 0.2 (10)	0.9 ± 0.2 (10)
GLU[8] (㎎/dℓ)	190 ± 66 (10)	178 ± 54 (10)	185 ± 41 (10)	217 ± 53 (10)
CHO[9] (㎎/dℓ)	101 ± 16 (10)	102 ± 16 (10)	112 ± 14 (10)	111 ± 18 (10)
TG[10] (㎎/dℓ)	82 ± 46 (10)	97 ± 42 (10)	85 ± 42 (10)	78 ± 35 (10)
PRO[11] (g/dℓ)	4.8 ± 0.2 (10)	4.8 ± 0.3 (10)	4.8 ± 0.2 (10)	5.0 ± 0.2 (10)
ALB[12] (g/dℓ)	1.7 ± 0.1 (10)	1.7 ± 0.1 (10)	1.7 ± 0.1 (10)	1.7 ± 0.1 (10)
A/G ratio[13]	0.55 ± 0.02 (10)	0.53 ± 0.02 (10)	0.54 ± 0.03 (10)	0.53 ± 0.02 (10)
LDH[14] (IU/L)	306 ± 193 (10)	291 ± 118 (10)	263 ± 155 (10)	271 ± 142 (10)
CPK[15] (U/L)	71 ± 34 (10)	83 ± 44 (10)	69 ± 20 (10)	62 ± 22 (10)
Ca[16] (㎎/dℓ)	9.7 ± 0.6 (10)	9.8 ± 0.4 (10)	9.7 ± 0.4 (10)	9.7 ± 0.6 (10)
IP[17] (㎎/dℓ)	9.8 ± 2.3 (10)	9.4 ± 1.7 (10)	10.5 ± 2.1 (10)	10.6 ± 2.6 (10)
Mg[18] (㎎/dℓ)	3.2 ± 0.6 (10)	3.1 ± 0.3 (10)	3.1 ± 0.3 (10)	3.1 ± 0.5 (10)
Na[19] (mmol/L)	154 ± 3 (10)	153 ± 2 (10)	153 ± 3 (10)	151* ± 2 (10)
K[20] (mmol/L)	4.8 ± 0.7 (10)	4.9 ± 0.5 (10)	4.9 ± 0.6 (10)	5.0 ± 0.5 (10)
Cl[21] (mmol/L)	114 ± 3 (10)	113 ± 2 (10)	115 ± 2 (10)	114 ± 2 (10)

Mean ± S.D (number of animals). 1: aspartate aminotransferase, 2: alanine aminotransferase, 3: alkaline phosphatase, 4 total bilirubin, 5: blood urea nitrogen, 6: creatinine, 7: uric acid, 8: glucose, 9: total cholesterol, 10: triglyceride, 11: total protein, 12: albumin, 13: albumin/globulin ratio, 14: lactate dehydrogenase, 15: creatine phosphokinase, 16: calcium, 17: inorganic phosphorus, 18: magnesium, 19: sodium, 20: potassium, 21: chloride. *: Significant difference compared to the control group value; $p < 0.05$.
